# Synthesis and antibacterial action of 3’,6’-disubstituted spectinomycins

**DOI:** 10.1038/s41429-024-00750-2

**Published:** 2024-06-18

**Authors:** Suresh Dharuman, Gregory A. Phelps, Christine M. Dunn, Laura A. Wilt, Patricia A. Murphy, Robin B. Lee, Hannah E. Snoke, Petra Selchow, Klara Haldimann, Erik C. Böttger, Sven N. Hobbie, Peter Sander, Richard E. Lee

**Affiliations:** 1 Department of Chemical Biology and Therapeutics, St. Jude Children’s Research Hospital, 262 Danny Thomas Place, MS#1000, Memphis, TN 38105 USA; 2 Graduate School of Biomedical Sciences, St. Jude Children’s Research Hospital, Memphis, TN 38103 USA; 3 Institute of Medical Microbiology, University of Zurich, Gloriastrasse 28/30, CH-8006 Zurich, Switzerland; 4 Division of Clinical Bacteriology and Mycology, University Hospital Basel, Petersgraben 4, CH-4031 Basel, Switzerland; 5 National Reference Center for Mycobacteria, Gloriastrasse 28/30, CH-8006 Zurich, Switzerland

**Keywords:** Antibiotics, Drug discovery and development

## Abstract

Spectinomycin is an aminocyclitol antibiotic with a unique ribosomal binding site. Prior synthetic modifications of spectinomycin have enhanced potency and antibacterial spectrum through addition at the 6’-position to produce trospectomycin and to the 3’-position to produce spectinamides and aminomethyl spectinomycins. This study focused on the design, synthesis, and evaluation of three 3’,6’-disubstituted spectinomycin analogs: trospectinamide, *N*-benzyl linked aminomethyl, and *N*-ethylene linked aminomethyl trospectomycins. Computational experiments predicted that these disubstituted analogs would be capable of binding within the SPC ribosomal binding site. The new analogs were synthesized from trospectomycin, adapting the previously established routes for the spectinamide and aminomethyl spectinomycin series. In a cell-free translation assay, the disubstituted analogs showed ribosomal inhibition similar to spectinomycin or trospectomycin. These disubstituted analogs demonstrated inhibitory MIC activity against various bacterial species with the 3’-modification dictating spectrum of activity, leading to improved activity against mycobacterium species. Notably, *N*-ethylene linked aminomethyl trospectomycins exhibited increased potency against *Mycobacterium abscessus* and trospectinamide displayed robust activity against *M. tuberculosis*, aligning with the selective efficacy of spectinamides. The study also found that trospectomycin is susceptible to efflux in *M. tuberculosis* and *M. abscessus*. These findings contribute to the understanding of the structure-activity relationship of spectinomycin analogs and can guide the design and synthesis of more effective spectinomycin compounds.

## Introduction

**Fig. 1:**
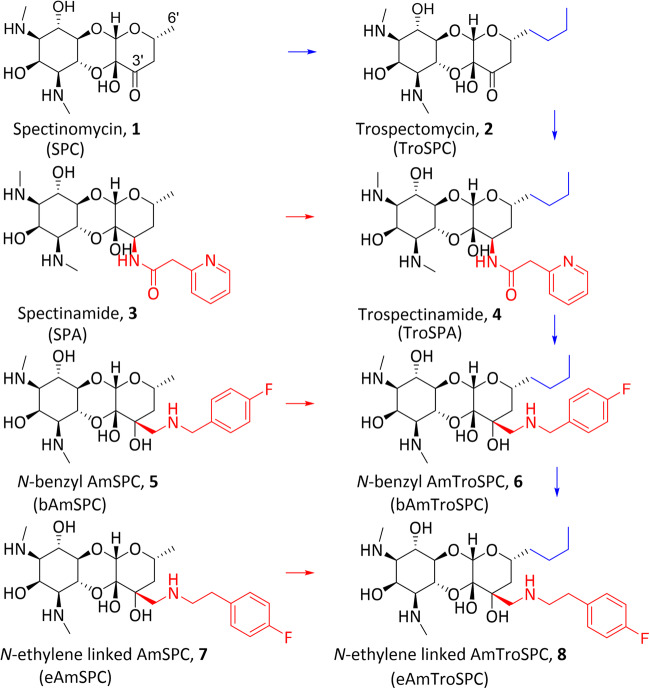
Analogs of spectinomycin and trospectomycin. Blue arrows highlight addition of 6’-propyl and red arrows highlight addition to 3’-modifications to the spectinomycin analogs

Many of our essential classes of antibiotics, such as beta-lactams, macrolides, and aminoglycosides, trace their origins back to natural products [1]. Through careful modifications, researchers have harnessed the power of semisynthetic chemistry to enhance the effectiveness, broaden the spectrum of activity, and overcome resistance mechanisms exhibited by drug-resistant pathogens [2]. An illustration of this concept can be seen in the strategic modifications applied to the natural product spectinomycin (SPC, **1** in Fig. 1), an aminocyclitol antibiotic produced by *Streptomyces spectabilis* historically used to treat *Neisseria gonorrhoeae* infections [3, 4]. SPC exerts its antimicrobial action by binding helix 34 of the 16S rRNA of the bacterial 30S ribosomal subunit thereby impeding bacterial translation [5]. SPC has a limited spectrum of activity due to innate resistance mechanisms in most organisms, largely mediated by efflux, and plasmid-encoded modifying enzymes prevalent in Gram-negative bacteria [6–9]. Semisynthetic modifications utilizing the SPC scaffold have successfully expanded the spectrum activity and improved antimicrobial activity for SPC analogs.

The most clinically advanced SPC modified analog, trospectomycin (TroSPC, **2** in Fig. 1), was discovered in the 1980’s and developed by the Upjohn company [10]. This 6’-propyl SPC analog displayed markedly increased activity, ranging from 2 to 50 times greater than SPC, against a diverse panel of microorganisms, including atypical bacteria, Gram-positive, Gram-negative, and non-tuberculous mycobacterium (NTM) species [11–13]. TroSPC showed favorable results in early clinical trials for sexually transmitted bacterial infections, but further trials were halted due to intense market competition.

Previous work by our group led to the development of three lead series of 3’-SPC analogs that overcome innate resistance to SPC in their relevant pathogens [14–19]. The spectinamides (SPA, **3** in Fig. 1) were developed first as a class of narrow-spectrum *Mycobacterium tuberculosis* agents that exert their improved action through avoidance of Tap-mediated (Rv1258c) efflux [14, 16]. *N*-benzyl substituted AmSPC (bAmSPC, **5** in Fig. 1) were developed against pathogens causing respiratory tract, biothreat, and sexually transmitted bacterial infections [15, 17, 18]. *N*-ethylene linked AmSPC (eAmSPC, **7** in Fig. 1), which differ from bAmSPC by an additional linker carbon between the aminomethyl and the terminal substitution, expanded the antimicrobial spectrum of the SPC class to broadly cover mycobacterial species, including the emerging NTM pathogen *M. abscessus* [20]. In *M. abscessus*, the increase in potency of eAmSPC is the result of greater accumulation, principally by avoiding high-level, TetV (Mab2780c) mediated efflux [20, 21].

**Scheme 1:**
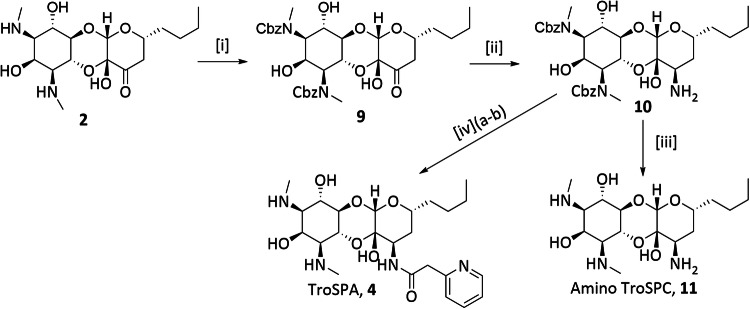
Synthesis of Amino TroSPC **11** and Trospectinamide **4**. Reagents and conditions: [i] CbzCl, NaHCO_3_, Acetone/H_2_O, 82%; [ii] NH_4_NO_3_, MeOH/AcOH, 2-picoline-borane complex, 32%; [iii] Pd/C, H_2_, MeOH, 69%; [iv] (a) 2-Pyridylacetic acid hydrochloride, HATU, DIPEA, DMF (b) Pd/C, H_2_, MeOH, 31% over 2 steps

**Scheme 2:**
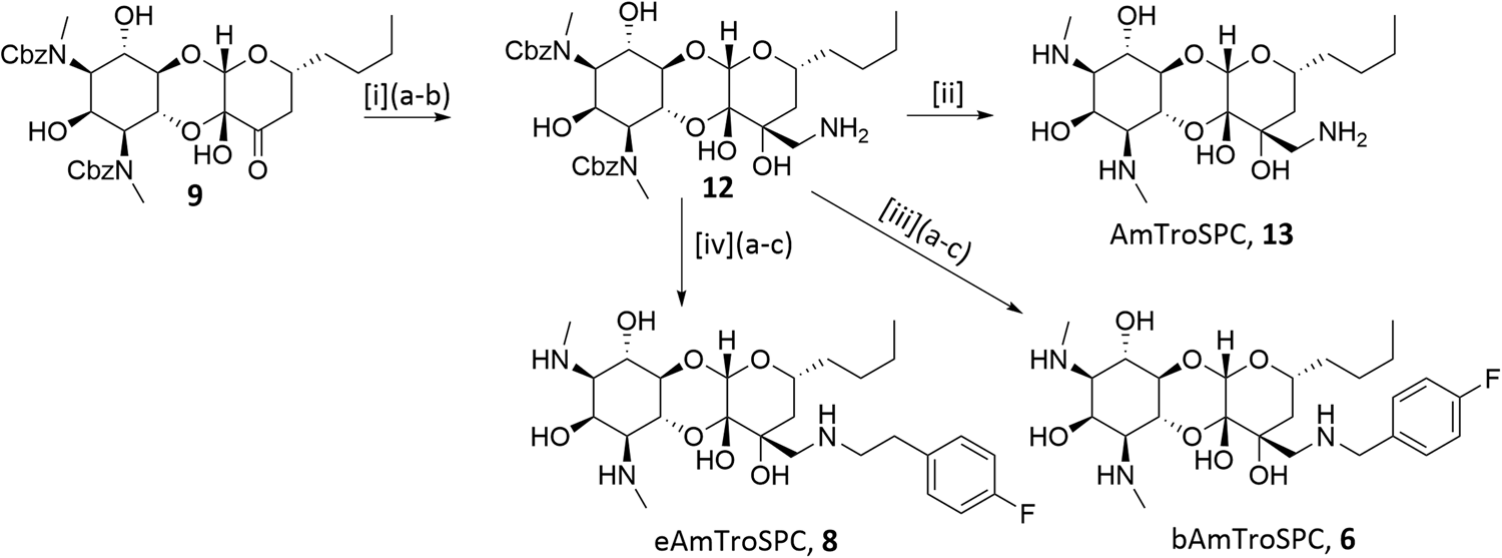
Synthesis of Aminomethyl TroSPC analogs **6,** **8**, and **13**. Reagents and conditions: [i] (a) KCN, 1 M AcOH in MeOH, (b) Raney Ni, H_2_, 2 M AcOH in MeOH, 29% over 2 steps; [ii] Pd/C, H_2_, MeOH, 65%; [iii] (a) 4-Fluoro-benzaldehyde, picoline-borane complex, MeOH/AcOH, (b) CbzCl, NaHCO_3_, Acetone/water, (c) Pd/C, H_2_, MeOH, 22% over 3 steps; [iv] (a) (4-Fluorophenyl)acetaldehyde, picoline-borane complex, MeOH/AcOH, (b) CbzCl, NaHCO_3_, Acetone/water, (c) Pd/C, H_2_, MeOH, 17% over 3 steps

Given TroSPC generally has improved antimicrobial activity compared to SPC [11–13], we hypothesized that combining the 6’-propyl side chain from TroSPC with the 3’-modifications from our lead spectinomycin analogs (**3,** **5**, and **7**) might result in improved antimicrobial activity. In this study, we report the synthesis and evaluation of three 3’,6’-disubstituted spectinomycin analogs: trospectinamide (TroSPA, **4** in Fig. 1), *N*-benzyl linked aminomethyl TroSPC (bAmTroSPC, **6** in Fig. 1) and *N*-ethylene linked aminomethyl TroSPC (eAmTroSPC, **8** in Fig. 1), as well as amino TroSPC (**11** in Scheme 1) and aminomethyl TroSPC (**13** in Scheme 2). These analogs exhibit comparable translational inhibitory properties against mycobacterial ribosomes, while closely retaining the antimicrobial characteristics of their corresponding 3’-SPC analogs. Our investigation also demonstrated that TroSPC is subject to efflux in *M. tuberculosis* and *M. abscessus* by Rv1258c and Mab2780c, respectively. This is consistent with SPC resistance mechanisms previously reported [8, 14, 21]. In *M. tuberculosis*, the 3’-modification was found to dominate the activity, as the efflux ratios of the disubstituted analogs were comparable to the single 3’-SPA and AmSPC substituted compounds. In *M. abscessus*, addition of the 6’-propyl side chain to SPC and SPA reduced Mab2780c mediated efflux 8-fold and abolished it completely when combined with the benzyl or ethylene 3’-aminomethyl substitution. However, this avoidance of efflux by Mab2780c did not result in increased antimicrobial activity over the parent SPC analogs, possibly due to reduced uptake or efflux by the Tap efflux pump homolog (Mab1409c). These results add to the structure-activity relationship of SPC analogs, aiding in the future design and synthesis of more efficacious compounds.

## Materials and methods

### Chemicals, reagents, and instrumental

All the reactions were carried out in oven-dried glassware under an atmosphere of nitrogen. All solvents and reagents were purchased from commercial vendors and used without further purification. All reagent-grade solvents used for chromatography were purchased from Fisher Scientific (Suwanee, GA), and flash column chromatography silica cartridges were obtained from Biotage Inc. (Lake Forest, VA). A Biotage FLASH column chromatography system was used to purify mixtures. All ^1^H NMR spectra were recorded on Bruker Avance500 (500 MHz, spectrometer). Chemical shifts (*δ*) are reported in parts per million, and coupling constants (J) are reported in hertz (Hz). All ^13^C NMR spectra were recorded on a Bruker Avance500 (125 MHz ^13^C) spectrometer. Chemical shifts (*δ*) are reported in parts per million. High-resolution mass spectra were recorded on a Waters Xevo G2 mass spectrometer. The purity of the compounds was determined using Waters Acquity UPLC-ELSD/SQD and was confirmed to be > 95%.

### Chemical synthesis

#### *N*,*N*’-Dibenzyloxycarbonyl-6’-propyl-spectinomycin (9)

TroSPC **2** was purchased from WuXi AppTec Co., Ltd. (Wuhan, China). To a solution of TroSPC **2** (0.40 g, 0.664 mmol) and NaHCO_3_ (0.223 g, 2.66 mmol) in H_2_O (4 ml) was added benzyl chloroformate (0.19 ml, 1.32 mmol, dissolved in 4 ml of acetone) dropwise under ice-bath. The reaction mixture was stirred for 1 h at room temperature and quenched with Conc. H_2_SO_4_ (pH 3) and the compound was extracted with EtOAc (2 ×30 ml). The combined organic layer was dried over Na_2_SO_4_ and concentrated to give crude product. The crude product was purified on silica gel flash column chromatography to yield **9** (0.35 g, 82%) as a white solid. ^1^H NMR (500 MHz, CD_3_OD, 1:1 ratio of rotamers) *δ* 7.46 – 7.15 (10H, m), 5.24 – 4.95 (4H, m), 4.70 (1H, br s), 4.63 – 4.50 (1H, m), 4.18 – 3.92 (5H, m), 3.63 (1H, m), 3.09 – 3.00 (6H, m), 2.80 – 2.61 (1H, m), 2.43 – 2.30 (1H, m), 1.75 – 1.57 (2H, m), 1.53 – 1.30 (4H, m), 0.93 (3H, td, *J* = 7.2, 2.9 Hz); ^13^C NMR (125 MHz, CD_3_OD) *δ* 157.5, 157.4, 157.0, 156.8, 136.9, 136.8, 128.2, 128.2, 128.2, 128.1, 127.6, 127.6, 127.6, 127.5, 127.4, 127.3, 127.3, 127.1, 96.8, 91.7, 74.0, 73.8, 73.4, 72.8, 71.3, 71.2, 67.0, 66.9, 66.8, 65.6, 65.5, 64.6, 64.5, 64.4, 64.3, 63.8, 60.2, 59.4, 56.6, 56.5, 43.5, 35.0, 30.5, 30.3, 30.2, 27.3, 27.0, 22.3, 22.2, 13.1, 13.0; HRMS (ESI) *m/z* calcd for C_33_H_43_N_2_O_11_ [M + H]^+^, 643.2866; found, 643.2844.

#### 3’-(*R*)-Amino-*N*,*N*’-dibenzyloxycarbonyl-3’-deoxy-6’-propyl-dihydrospectinomycin (10)

Ammonium nitrate (0.311 g, 3.89 mmol) and 2-picoline borane complex (42.0 mg, 0.389 mmol) were added to a solution of ketone **9** (0.250 g, 0.389 mmol) dissolved in MeOH (2.5 ml) and AcOH (0.25 ml) at room temperature and stirred for 2 h. Evaporated the reaction mixture and the compound was extracted with 1 M HCl (10 ×20 ml) to give pure amine and impurities were gone into EtOAc layer. The aqueous solution was neutralized with NaHCO_3_, and the free amine was extracted with EtOAc (1 ×50 ml), dried over Na_2_SO_4_ and concentrated to give pure amine **10** (80 mg, 32%) as a white solid. ^1^H NMR (400 MHz, CD_3_OD) *δ* 7.47 – 7.13 (10H, m), 5.28 – 4.99 (4H, m), 4.81 (1H, s), 4.60 (2H, s), 4.24 – 3.80 (5H, m), 3.21 – 3.12 (1H, m), 3.12 – 2.96 (6H, m), 1.93 (1H, br s), 1.68 – 1.40 (3H, m), 1.41 – 1.27 (4H, m), 0.91 (3H, ddd, *J* = 7.4, 4.8, 2.4 Hz); ^13^C NMR (100 MHz, CD_3_OD) *δ* 158.9, 158.4, 142.7, 138.2, 129.6, 129.5, 129.4, 129.3, 129.1, 128.8, 128.7, 128.7, 128.6, 128.5, 128.3, 128.0, 97.1, 95.4, 95.0, 75.0, 68.3, 65.3, 61.0, 58.0, 58.0, 36.1, 31.8, 28.7, 23.7, 14.4. HRMS (ESI) *m/z* calcd for C_33_H_46_N_3_O_10_ [M + H]^+^, 644.3183; found, 644.3174.

#### 3′-Deoxy -3′(*R*)-[(pyridin-2-yl) acetylamino]-6’-propyl-dihydrospectinomycin dihydrochloride (4)

The amine **10** (30 mg, 0.047 mmol) was dissolved in DMF (1.0 ml) and treated with 2-pyridylacetic acid hydrochloride (10 mg, 0.056 mmol), HBTU (21 mg, 0.056 mmol), and *N*, *N*’-diisopropylethylamine (25 μl, 0.140 mmol) at room temperature. After 15 min, the reaction was quenched with water (5 ml), extracted with EtOAc (3 ×10 ml), dried over Na_2_SO_4_, and concentrated. The crude product was purified on silica gel column chromatography (Eluents: 80-100% EtOAc in hexane) to give amide compound. To an amide (12 mg, 0.016 mmol) in MeOH (2 ml) was added 1 M HCl in MeOH (63 μl) and Pd/C (3 mg). The reaction mixture was hydrogenated under H_2_ atmosphere for overnight. Evaporated the reaction mixture and triturated with acetone to give pure **4** (8.0 mg, 31% over 2 steps) as a white solid. ^1^H NMR (500 MHz, D_2_O) *δ* 8.65 (1H, d, *J* = 5.9 Hz), 8.46 (1H, t, *J* = 8.0 Hz), 7.95 – 7.75 (2H, m), 4.91 (1H, s), 4.68 (1H, s), 4.30 (1H, t, *J* = 10.5 Hz), 4.12 (2H, d, *J* = 4.6 Hz), 3.90 (3H, dq, *J* = 28.7, 9.1, 8.4 Hz), 3.43 (1H, dd, *J* = 11.2, 2.8 Hz), 3.18 (1H, dd, *J* = 10.2, 2.7 Hz), 2.78 – 2.76 (1H, m), 2.74 (6H, d, *J* = 2.8 Hz), 1.84 (1H, d, *J* = 15.4 Hz), 1.68 (1H, d, *J* = 14.4 Hz), 1.50 (2H, ddd, *J* = 31.1, 14.1, 7.0 Hz), 1.35 – 1.13 (4H, m), 0.79 (3H, t, *J* = 6.8 Hz); ^13^C NMR (125 MHz, D_2_O) *δ* 169.0, 149.3, 146.8, 141.5, 128.2, 125.7, 93.1, 90.2, 71.5, 69.9, 65.9, 65.6, 61.4, 59.8, 58.4, 52.2, 39.3, 33.6, 32.1, 30.6, 30.5, 26.4, 21.9, 13.2; HRMS (ESI) *m/z* calcd for C_24_H_39_N_4_O_7_ [M + H]^+^, 495.2818; found, 495.2801.

#### 3’-(*R*)-Amino-3’-deoxy-6’-propyl-dihydrospectinomycin trihydrochloride (11)

A mixture containing 3’-(*R*)-amino-*N*,*N*’-dibenzyloxycarbonyl-3’-deoxy-6’-propyl-dihydrospectinomycin **10** (25 mg), Pd/C (10 mg) in methanol (2 ml), and 1 M HCl in methanol (150 µl) underwent hydrogenation under a hydrogen atmosphere (H_2_ balloon) overnight. Following the reaction, the catalyst was removed by filtration, and the resulting filtrate was evaporated to obtain the crude product. The crude product was then subjected to trituration with acetone, yielding pure **11** (12 mg, 69%) as a white solid. ^1^H NMR (400 MHz, D_2_O) *δ* 5.06 (1H, s), 4.38 (1H, t, *J* = 10.7 Hz), 4.18 – 3.87 (3H, m), 3.71 (1H, s), 3.57 (1H, d, *J* = 11.5 Hz), 3.43 – 3.18 (2H, m), 2.86 (6H, s), 2.19 – 1.78 (2H, m), 1.72 – 1.56 (2H, m), 1.41 – 1.30 (4H, m), 0.91 (3H, t, *J* = 6.7 Hz); ^13^C NMR (100 MHz, D_2_O) *δ* 92.5, 70.3, 69.8, 66.0, 65.9, 61.4, 59.9, 58.3, 52.8, 33.6, 30.8, 30.6, 30.2, 26.3, 21.9, 13.2; HRMS (ESI) *m/z* calcd for C_17_H_34_N_3_O_6_ [M + H]^+^, 376.2447; found, 376.2432.

#### 3’-(*R*)-Aminomethyl-*N*,*N*’-dibenzyloxycarbonyl-6’-propyl-dihydrospectinomycin (12)

To a solution of Cbz-TroSPC (300 mg, 0.47 mmol) 1 M AcOH in MeOH (5 ml) was added KCN (72 mg in 1 ml of water) at room temperature and the reaction mixture was stirred for 30 min. Evaporated the reaction mixture and diluted in EtOAc (2 ×20 ml), washed with water. The organic layer was dried over Na_2_SO_4_ and concentrated. To a solution of crude cyanohydrin (250 mg, 0.37 mmol) in 2 M AcOH in MeOH (3 ml) was added Raney Ni (freshly washed, crude wt = 500 mg) and stirred under H_2_ atm for 16 h. Filtered off the catalyst and filtrate was evaporated. The crude was dissolved in EtOAc, the compound was extracted with 0.1 M HCl in water (5 ×10 ml) as HCl salt. The water layer quenched with solid NaHCO_3_ and NH_3_ in MeOH to give pH 8. The amino compound was re-extracted from water using EtOAc (1 ×25 ml), dried over Na_2_SO_4_ and concentrated to give **12** (90 mg, 29% over 2 steps) as a white solid. ^1^H NMR (500 MHz, CD_3_OD) *δ* 7.44 – 7.19 (10H, m), 5.29 – 4.94 (4H, m), 4.72 (1H, d, *J* = 6.7 Hz), 4.60 (2H, s), 4.17 – 3.87 (1H, m), 3.66 – 3.48 (1H, m), 3.22 – 3.16 (1H, m), 3.09 (6H, s), 2.93 – 2.78 (1H, m), 1.82 – 1.41 (4H, m), 1.41 – 1.25 (3H, m), 0.97 – 0.84 (3H, m); ^13^C NMR (125 MHz, CD_3_OD) *δ* 157.5, 157.0, 157.0, 141.3, 136.8, 128.3, 128.2, 128.1, 128.0, 127.7, 127.6, 127.5, 127.4, 126.9, 126.6, 94.9, 93.1, 73.4, 70.4, 66.9, 63.8, 59.4, 56.7, 48.2, 48.0, 47.8, 47.7, 47.5, 47.3, 47.1, 43.7, 38.4, 34.9, 30.4, 27.2, 22.4, 13.1; HRMS (ESI) *m/z* calcd for C_34_H_48_N_3_O_11_ [M + H]^+^, 674.3288; found, 674.3313.

#### 3′(*R*)-[(4-Fluorobenzyl)aminomethyl]-6’-propyl-dihydrospectinomycin trihydrochloride (6)

A stirred solution of 3’-(*R*)-aminomethyl-*N*,*N*’-dibenzyloxycarbonyl-6’-propyl-dihydrospectinomycin **12** (50 mg, 0.074 mmol) in MeOH:AcOH (10:1) (2 ml) was prepared, and 4-fluorobenzaldehyde (19 mg, 0.148 mmol) along with 2-picoline borane (8 mg, 0.074 mmol) were added. The mixture was stirred at room temperature for 1 h. After removal of methanol, the residue was partitioned between EtOAc and water, and the two-phase mixture was extracted with EtOAc (2 ×5 ml). The combined organic layers were dried with Na_2_SO_4_ and concentrated under reduced pressure, yielding crude amine. To this crude amine (50 mg, 0.064 mmol) in H_2_O and acetone (2 ml), NaHCO_3_ (21 mg, 0.256 mmol), and benzyl chloroformate (27 μl, 0.192 mmol) were added under an ice bath. The reaction mixture was stirred for 1 h at room temperature, quenched with water, and extracted with EtOAc (2 ×10 ml). After drying the combined organic layers with Na_2_SO_4_ and concentrating under reduced pressure, column chromatography (Eluents: 60 – 80% EtOAc in hexane) was performed to obtain the corresponding Cbz-protected amine. The Cbz-protected amine was dissolved in MeOH (2 ml) and 1 M HCl in MeOH (90 μl) and stirred under an H_2_ atmosphere in the presence of Pd/C (5 mg) overnight. The catalyst was filtered off, and the filtrate was evaporated to give the crude product, which was triturated with acetone to yield **6** (10 mg, 22% over 3 steps) as a colorless solid. ^1^H NMR (500 MHz, D_2_O) *δ* 7.53 (2H, dd, *J* = 8.2, 5.3 Hz), 7.24 (2H, t, *J* = 8.6 Hz), 4.76 (1H, s), 4.35 – 4.25 (3H, m), 4.05 – 3.95 (2H, m), 3.62 – 3.49 (2H, m), 3.42 (1H, d, *J* = 13.6 Hz), 3.26 – 3.19 (2H, m), 2.83 (6H, s), 2.81 – 2.76 (1H, m), 1.87 (1H, t, *J* = 12.7 Hz), 1.76 (1H, d, *J* = 13.8 Hz), 1.54 (2H, qt, *J* = 14.6, 8.7 Hz), 1.31 (4H, dt, *J* = 24.6, 6.9 Hz), 0.87 (3H, d, *J* = 6.9 Hz); ^13^C NMR (125 MHz, D_2_O) *δ* 164.2, 162.3, 132.3, 132.2, 126.0, 125.9, 116.3, 116.1, 93.3, 92.5, 72.2, 70.7, 69.6, 65.9, 65.3, 61.5, 59.4, 58.5, 50.7, 48.8, 38.2, 33.8, 30.5, 30.3, 26.2, 21.9, 13.2; HRMS (ESI) *m/z* calcd for C_25_H_41_FN_3_O_7_ [M + H]^+^, 514.2928; found, 514.2913.

#### 3′(*R*)-[(4-Fluorophenylethyl)aminomethyl]-6’-propyl-dihydrospectinomycin trihydrochloride (8)

Compound **8** (8.0 mg, 17% over 3 steps) was synthesized analogously as **6** from **12** using (4-fluorophenyl)acetaldehyde, as a white solid. ^1^H NMR (500 MHz, D_2_O) *δ* 7.35 – 7.33 (2H, m), 7.17 – 7.13 (2H, m), 4.88 (1H, s), 4.29 (1H, t, *J* = 10.3 Hz), 4.06 – 3.95 (2H, m), 3.74 – 3.64 (1H, m), 3.53 (2H, t, *J* = 12.3 Hz), 3.45 – 3.21 (4H, m), 3.30 – 3.05 (3H, m), 2.83 (6H, s), 1.90 (1H, t, *J* = 12.7 Hz), 1.80 (1H, d, *J* = 13.8 Hz), 1.59 (2H, s), 1.32 (4H, s), 0.89 (3H, d, *J* = 7.3 Hz); ^13^C NMR (125 MHz, D_2_O) *δ* 162.8, 160.8, 132.3, 132.2, 132.0, 132.0, 130.5, 130.4, 116.3, 116.1, 115.8, 115.6, 93.3, 92.6, 72.2, 70.7, 69.6, 65.9, 65.3, 61.5, 59.5, 58.5, 50.4, 49.4, 38.5, 33.8, 30.5, 30.4, 30.3, 26.3, 21.9, 13.2; HRMS (ESI) *m/z* calcd for C_26_H_43_FN_3_O_7_ [M + H]^+^, 528.3085; found, 528.3061.

#### 3’-(*R*)-Aminomethyl-6’-propyl-dihydrospectinomycin (13)

A solution of 3’-(*R*)-aminomethyl-*N*,*N*’-dibenzyloxycarbonyl-6’-propyl-dihydrospectinomycin **12** (30 mg, 0.045 mmol) in MeOH (2 ml) was prepared, and Pd/C (10 mg) and 1 M HCl in MeOH (0.178 ml, 0.178 mmol) were added at room temperature. The reaction mixture was hydrogenated for 30 min under an H_2_ atmosphere (H_2_ balloon). After filtering off the excess catalyst, the solution was evaporated, and the resulting residue was washed with acetone (5 ml) and EtOAc (5 ml), yielding pure product **13** (15 mg, 65%) as a white solid. ^1^H NMR (500 MHz, D_2_O) *δ* 4.86 (1H, s), 4.74 (1H, s), 4.22 (1H, t, *J* = 10.4 Hz), 3.97 (1H, t, *J* = 10.4 Hz), 3.89 (1H, t, *J* = 10.2 Hz), 3.67 (1H, dt, *J* = 12.4, 6.2 Hz), 3.55 – 3.34 (2H, m), 3.26 – 3.03 (2H, m), 2.75 (6H, s), 1.83 (1H, t, *J* = 13.0 Hz), 1.72 (1H, d, *J* = 13.8 Hz), 1.57 – 1.46 (2H, m), 1.34 – 1.16 (4H, m), 0.80 (3H, t, *J* = 7.1 Hz); ^13^C NMR (125 MHz, D_2_O) *δ* 93.3, 92.8, 72.0, 70.8, 69.6, 65.9, 65.2, 61.5, 59.5, 58.5, 42.4, 38.1, 33.9, 30.5, 30.3, 26.2, 21.9, 13.2; HRMS (ESI) *m/z* calcd for C_18_H_36_N_3_O_7_ [M + H] + , 406.2553; found, 406.2542.

### Molecular docking/molecular dynamics simulation

The molecular docking and molecular dynamics (MD) studies were performed on SPC **1**, TroSPC **2**, TroSPA **4**, and eAmTroSPC **8** using a model of the Mycobacterial ribosomal RNA/RpsE complex as previously described [19]. Briefly, ligands were prepared in Schrödinger and docked onto the prepped rRNA/RpsE complex using default settings, with constraints as previously described [19, 22]. The highest-ranked docked poses evaluated by Glide score of SPC **1**, TroSPC **2**, Trospectinamide **4**, and the *N*-ethylene linked AmTroSPC **8** were advanced to MD simulations using Amber22 [23]. The rRNA/RpsE complex was parameterized using the ff14SB and OL3 forcefield, and the general Amber force field was applied to the ligands [19]. The rRNA/RpsE/ligand complexes were neutralized with sodium ions and solvated with TIP3P waters in an octahedron box with a 10 Å boundary [19]. The complex was energy minimized for 1000 steps while fixed, 2500 steps without restraints, heated to 300 K over 250,000 steps for 500 ps with weak restraints, and then subject to 20 ns with the NPT ensemble at 300 K with terminal residues harmonically restrained. The energetic contributions were calculated by MM/GBSA method using 1000 frames from the final 2 ns of the simulation, followed by free energy decomposition for per-residue analysis[19, 22, 23].

### Bacterial strains and growth conditions

Bacterial strains were obtained through ATCC, BEI resources, or from academic laboratories. *Acinetobacter baumannii* (ATCC 19606), *Enterococcus faecalis* (ATCC 29212), *E. faecium* (ATCC 19434), *Escherichia coli* (K12), *Klebsiella pneumoniae* (ATCC 33495), *Pseudomonas aeruginosa* (PA01), *Staphylococcus aureus* (USA300_FPR3757), and *S. epidermidis* (ATCC 14990) were maintained in Mueller Hinton (MH) (Fisher Scientific) broth or agar plates. *E. coli* JW5503 *ΔtolC* (*ΔtolC*:Kan) was maintained on MH broth or agar containing 25 µg ml^−1^ kanamycin. *Streptococcus pneumoniae* (R6) and *S. pyogenes* (ATCC 700294) were cultured in MH in the presence of 10% (v/v, final) defibrinated, lysed horse blood (LHB). *Mycobacterium smegmatis* (ATCC 700084, mc(2) 155), *M. abscessus* (ATCC 19977), and *M. abscessus* ∆*2780c* were maintained in cation-adjusted MH2 (Fisher Scientific) broth and agar [20]. *M. tuberculosis* (H37Rv) and *M*. *tuberculosis* ∆1258c was cultured in Middlebrook 7H9 broth (Difco Laboratories) supplemented with 10% albumin-dextrose complex and 0.05% (v/v) Tween 80 (pH 7.4).

### Antibacterial susceptibility testing

Minimal inhibitor concentration (MIC) assays were determined using broth microdilution in appropriate media (indicated above) according to Clinical Laboratory Standards Institute (CLSI; M24 for Mycobacterium, M100 for all other bacteria tested) with the exception that *Streptococcus spp*. MICs, which were performed without the addition of lysed horse blood to media. MICs were conducted using twofold serial dilutions in 96-well plates (Thermo Fisher Scientific, cat. 163320), starting at a drug concentration of at least 64 µg ml^−1^ and a final volume of 0.2 ml. Plates were incubated in 5% CO_2_ at 37 °C and MICs were recorded by visual inspection after 16 to 20 h of incubation, except for mycobacterial MICs, which were recorded after 3 days for rapidly growing Mycobacteria and 7 days for *M. tuberculosis*. Mycobacterial MICs were performed in biologic triplicate, whereas all other MICs were performed in duplicate. Median or range of MIC values are reported.

### Ribosomal inhibition assays

Mycobacterial cell-free translation inhibition assays were performed as described previously [24]. In brief, *M. smegmatis* strain SZ380 bacterial cells were disrupted and emulsified with a microfluidizer processor (Microfluidics, Westwood, MA, USA) at 25,000 lb/in^2^. S30 extracts were prepared by addition of dithiothreitol (DTT) to 1 mM and centrifugation at 30,000 × *g* at 4 °C. Translation reaction mixtures containing either test article or DMSO vehicle control and 4 μl of the S30 extract, 0.2 mM amino acid mix, 6 μg tRNA (Sigma), 0.4 μg hFluc mRNA, 0.3 μl protease inhibitor (complete, EDTA-free, Roche), 12 U RNAse inhibitor (Ribolock, Thermo Scientific), 6 μl S30 premix without amino acids (Promega), plus water to a final reaction volume of 15 μl were incubated for 1 h at 37 °C. The reaction was stopped on ice before adding 75 μl of luciferase assay reagent (Promega) and recording of luminescence. Regression analysis and IC_50_ calculations were performed using GraphPad Prism version 9.3.1 by using the equation [log(inhibitor) vs. response–Variable slope (four parameters)] with the fitting method: least squares (ordinary) fit. Y = Bottom + (Top-Bottom)/(1 + 10^((X-LogIC_50_))).

## Results and discussion

### Computer aided design of 3’,6’-disubstituted spectinomycin analogs

**Fig. 2:**
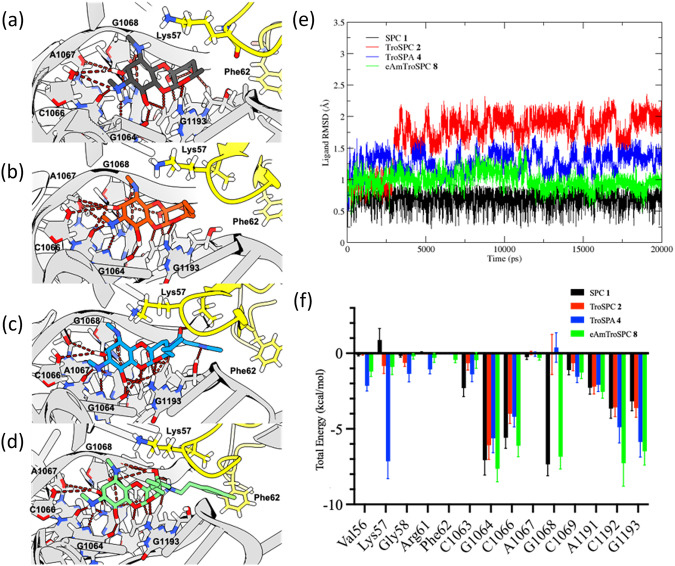
Modeled binding poses of SPC **1** in black (**a**), TroSPC **2** in red (**b**), TroSPA **4**in blue (**c**), and *N*-ethylene linked AmTroSPC **11**in green (**d**). Images are from the final frame of a 20 ns MD simulation in AMBER22 (**e**, **f**). Hydrogen bond interactions with nucleic acids G1064, C1066, A1067, G1068, A1191 and G1193 are shown in red and with Lys57 highlighted in yellow. MD simulation trajectory for a 20 ns MD simulation with protein backbone RMSD (**e**) and ligand position RMSD (**f**) for each docked system

Based on the improved antimicrobial activity of TroSPC against various pathogens when compared to SPC (11–13), we hypothesized that combining the 6’-propyl side chain from TroSPC **2** with our lead spectinomycin analogs (**3, 5**, and **7**) might further improve their antibacterial activity. To see if this is feasible, we first examined ribosome binding of TroSPC **2** using in silico experiments comparing SPC **1** and TroSPC **2** to model how the 6’-propyl side chain impacts ribosomal binding at the helix 34 SPC binding site as there are no current crystal structures of TroSPC **2** bound to a bacterial ribosome. Molecular docking showed that TroSPC **2** occupies the SPC binding site with an RMSD of 1.5 Å between the two structures, consistent with their similar glide docking scores of −9.96 kcal mol^−1^ and −10.05 kcal mol^−1^, respectively (Fig. 2a, b, Table 1). Throughout a 20 ns MD simulation, SPC **1** was not perturbed from the binding pocket and maintained hydrogen bonding interactions with no large disturbances in the ligand RMSD (Fig. 2e, black). MD simulation of TroSPC **2** maintained the same hydrogen bonding interactions that drive SPC **1** recognition (Fig. 2f, red). To assess the feasibility of 3’-modifications to TroSPC **2**, docking and MD simulations were repeated for the candidate molecules TroSPA **4** and eAmTroSPC **8** (Fig. 2e, blue and green respectively). Modeling of these compounds showed consistent binding poses compared to the SPC **1** and TroSPC **2**, indicating that additions to the 3’-position of the TroSPC **2** molecule can be well tolerated for binding (Fig. 2c–f).

**Table 1:** Glide Score of docked structures from Schrödinger, and calculated binding free energy onto the Mycobacteria rRNA/RpsE complex using MM/GBSA calculated from the last 2 ns of MD simulation in AMBER22

Compound	Glide score (kcal/mol)	E_solv_^a^ (kcal/mol)	E_VDW_^a^ (kcal/mol)	∆G_total_^a^ (kcal/mol)
SPC (**1**)	−9.96	−5.08 ± 0.13	−53.06 ± 3.5	−82.13 ± 3.6
TroSPC (**2**)	−10.0	−5.54 ± 0.15	−51.21 ± 3.5	−60.10 ± 3.9
TroSPA (**4**)	−11.8	−7.60 ± 0.16	−73.14 ± 4.1	−88.17 ± 4.5
eAmTroSPC (**8**)	−11.7	−7.60 ± 0.24	−74.97 ± 4.3	−109.76 ± 5.3

^a^Values represent an average over 1000 MD frames ± standard deviation

Like SPC, modeling of TroSPC **2**, TroSPA **4**, and eAmTroSPC **8** maintained hydrogen bonding interactions with key residues in the helix 34 RNA loop, and 3’-modifications were able to form additional hydrogen bonding interactions with the ribosomal RNA/RpsE complex, reinforcing the feasibility of modifications to this region of the molecule (Fig. 2a–d). To better understand the contributions of additional interactions formed by modifications to the SPC **1** core, the free energy of binding for each ligand was determined using MM/GBSA using the last 1000 frames of each 20 ns MD simulation, and an energy decomposition analysis was performed on the binding site (Table 1, Fig. 2f). SPC **1** maintains a more stable position in the ribosome binding site across the MD simulation which may explain the more favorable *Δ*G_total_ compared to TroSPC **2**. However, the energy contributions from E_VDW_ and E_solv_ are comparable between the two molecules (Table 1). The 6’-propyl position of TroSPC **2** shows new interactions with the Lys57 and Gly58 residues within the RpsE protein. These interactions were maintained or improved upon in the MD simulations of the larger TroSPA **4** and eAmTroSPC **8** ligands (Fig. 2f). Additionally, TroSPA **4** and eAmTroSPC **8** formed new hydrogen bonding interactions with the RpsE protein backbone and C1192 or G1193 respectively (Fig. 2c, d, and f). Both ligands show more favorable energies (Table 1) and more consistent binding positions across the 20 ns MD simulation (Fig. 2e) compared to TroSPC **2**. While it is important to note that larger molecules can rank more favorably by computational scoring methods, the energy contributions from binding site interactions indicated additions to the TroSPC **2** core could improve hydrogen bonding interactions, stabilize the position of the 6’-propyl chain, and have the possibility to form new residue interactions with the RpsE protein (Fig. 2f). Therefore, computational docking indicated 3’-modifications to TroSPC **2** would be feasible to maintain ribosomal binding, and hybrid analogs were advanced for synthesis.

### Synthesis of 3’,6’-disubstituted SPC analogs

For the generation of TroSPC **2** at scale, the key intermediate in the synthesis of disubstituted analogs, we followed the previously published approach, transforming a protected spectinomycin-derived silyl enol ether into an enone through oxidation with *tert*-butyl hydroperoxide, followed by γ-alkylation using lithium bis(trimethylsilyl)amine and allyl chloride [25]. The synthesis of TroSPA **4** followed Scheme 1, TroSPC **2** was converted to Cbz TroSPC **9** using benzyl chloroformate and sodium bicarbonate. Treatment of **9** with NH_4_NO_3_ in the presence of a picoline-borane complex yielded amino Cbz TroSPC **10**. Coupling of **10** with 2-pyridyl acetic acid, in the presence of HATU and DIPEA, followed by hydrogenolysis, produced TroSPA **4**. Additionally, amino TroSPC **11** was synthesized via the hydrogenolysis of **10**.

The synthesis of aminomethyl TroSPC analogs followed Scheme 2. **9** was converted into a cyanohydrin, followed by Raney Ni reduction, resulting in aminomethyl Cbz TroSPC **12**. A reductive amination of **12** with 4-fluoro-benzaldehyde and subsequent hydrogenolysis yielded bAmTroSPC **6**. eAmTroSPC **8** was synthesized similarly, substituting 4-fluoro-benzaldehyde with 4-fluorophenyl-acetaldehyde. Aminomethyl TroSPC **13** was synthesized from intermediate **12** via hydrogenolysis reaction of Cbz groups.

### Ribosomal activity and mycobacterial susceptibility of the 3’,6’-disubstituted SPC analogs

The newly synthesized analogs were evaluated for their ability to inhibit ribosomal translation using *M. smegmatis* ribosomes in a cell-free, in vitro translation assay. The results from this assay (Table 2) showed that the combination of the 6’-propyl side chain with 3’-SPA or AmSPCs did not augment interaction with the ribosomal target. 3’-SPC analogs SPA **3**, bAmSPC **5** and eAmSPC **7** all have ribosomal inhibition IC_50_ values between 1.01 and 1.8 µM. Their corresponding 3’,6’-disubstituted SPC analogs (**4,** **5**, and **6**, respectively) had similar IC_50_ values (Ribo IC_50_ 2.31–2.49 µM). Compound **11**, the Amino TroSPC with a free amine group at the 3’-position, had ribosomal inhibition (Ribo IC_50_ 0.64 µM) corresponding to TroSPC **2** (Ribo IC_50_ 0.51 µM). However, the aminomethyl TroSPC **13** ribosomal inhibition IC_50_ value (Ribo IC_50_ 2.71 µM) corresponded with the benzyl and ethylene-linked AmSPC and AmTroSPC analogs. As expected, ribosomal inhibition did not correlate directly to whole-cell activity in *M. smegmatis*, as other factors contribute to MIC activity. The 3’-side chain in SPA and eAmSPC analogs has previously been shown to overcome innate efflux resistance mechanisms in *M. tuberculosis* and *M. abscessus*, and this was expected for the 3’,6’-disubstituted SPC analogs as well.

**Table 2:** Antibacterial susceptibility (MIC, µg ml^−1^), efflux ratios, and ribosomal affinity IC_50_ (µM) against Mycobacterial species

	*M. tuberculosis H37Rv*			*M. abscessus* ATCC 19977			*M. smegmatis*^a^	
Compound	WT MIC (µg ml^−1^)	*∆1258c* MIC (µg ml^−1^)	Efflux Ratio	WT MIC (µg ml^−1^)	*∆2780c* MIC (µg ml^−1^)	Efflux Ratio	WT MIC (µg ml^-1^)	Ribo. IC_50_ (µM)
SPC (**1**)	64	4	16	512	16	32	64	0.80^b^
TroSPC (**2**)	32	2	16	64	16	4	32	0.51
SPA (**3**)	2	4	0.5	64^c^	16^c^	4^c^	ND	1.80^d^
TroSPA (**4)**	2	1	2	64	16	4	32	2.45
bAmSPC (**5**)	16	2	8	128	8	16	ND	1.18^b^
bAmTroSPC (**6**)	8	1	8	64	64	1	64	2.49
eAmSPC (**7**)	2	0.5	4	4	1	4	4	1.01^b^
eAmTroSPC **(8)**	2	0.25	8	8	8	1	8	2.31
Amino TroSPC (**11**)	16	2	8	64	64	1	32	0.64
AmTroSPC (**13**)	64	8	8	64	64	1	128	2.71

^a^MIC assays were conducted using *M. smegmatis* strain MC^2^ 155. Ribosome inhibition assays were conducted using ribosomes purified from *M. smegmatis* strain SZ380

^b^Ribosomal IC_50_ for compounds **1,** **5**, and **7** were previously reported in ref. [20]

^c^Lead SPA 1810 (Compound 39 from ref. [16]) was substituted for SPA **3**. SPA 1810 possess an additional hydroxyl on the para position of the pyridine ring when compared to SPA **3**

^d^Ribosomal IC_50_ used for compound **3** was converted from µg/mL to µM from what was previously reported in ref. [16]

We then proceeded to test the analogs against clinically relevant Mycobacterium species, including *M. tuberculosis* and *M. abscessus* (Table 2). Considering that susceptibility to SPC and its analogs can be affected by the presence of efflux pumps in *M. tuberculosis* (Tap, Rv1258c) and *M. abscessus* (TetV, Mab2780c) [8, 21], we also assessed the compounds against isogenic deletion strains of these pumps in their respective organisms.

In *M. tuberculosis*, SPC has a relatively high MIC (64 µg ml^−1^) because it is subject to efflux by the MFS efflux pump Rv1258c. In this study, we find that TroSPC is also a substrate for Rv1258c. This was determined by calculating the efflux ratio between the wild-type (WT) and ∆*1258c* strains (MIC WT divided by MIC ∆*1258c)* (Table 2). SPC and TroSPC both have efflux ratios of 16. For the disubstituted analogs in this study, the 3’-modification was found to dominate the activity, as the MIC values and efflux ratios of the disubstituted analogs were comparable to the single 3’-SPA and AmSPC substituted compounds. TroSPA **4** and eAmTroSPC **8** were the most potent TroSPC analogs tested against WT *M. tuberculosis*, exerting an MIC of 2 µg ml^−1^. All other analogs had activities ranging from 8 to 64 µg ml^−1^. In line with previous structure activity relationship (SAR) observations, we observe that the 2-pyridyl moiety is key to avoiding Rv1258c-mediated efflux in *M. tuberculosis*, as only TroSPA **4** was found to be minimally impacted by Rv1258c status (efflux ratio = 2) [16]. All other TroSPC analogs had an 8-fold increase in susceptibility upon Rv1258c deletion, including eAmTroSPC **8**, with an MIC of 0.25 µg ml^−1^ in the ∆*1258c* strain.

In *M. abscessus*, SPC has a very high MIC (512 µg ml^−1^) and is subject to efflux by the MFS efflux pump Mab2780c. The susceptibility of *M. abscessus* to TroSPC was also found to be affected by Mab2780c, albeit to a lesser extent compared to SPC, with an efflux ratio of 4 vs 32, respectively (Table 2). Both SPC and TroSPC had MICs of 16 µg ml^−1^ in the ∆*2780c* strain, reinforcing that the expression of Mab2780c in *M. abscessus* plays an important role in resistance of spectinomycins. For the disubstituted analogs, the 3’-modification was again found to dominate the MIC activity, as the MIC values of the disubstituted analogs were comparable to the single 3’-SPA and AmSPC substituted compounds. Only eAmTroSPC **8** exhibited good whole cell activity against WT *M. abscessus* (MIC 8 µg ml^−1^). This finding is consistent with our previous report identifying the *N*-ethylene linkage of the aminomethyl group as critical in the enhanced activity of AmSPC against *M. abscessus* [20]. All other TroSPC analogs demonstrated low activity with MICs of 64 µg ml^−1^ or greater. Surprisingly, in *M. abscessus* combining the 6’-propyl group with bAmSPC **5** and eAmSPC **7** abolished efflux by Mab2780c. Compared to bAmSPC **5** and eAmSPC **7** with efflux ratios of 16 and 4, respectively, addition of the 6’-propyl group resulted in efflux ratios equal to 1 for bAmTroSPC **6** and eAmTroSPC **8**. This was unexpected given that TroSPC and the AmSPCs are known to be substrates for Mab2780c efflux [20]. The TroSPA **4** analog was still subject to efflux with an efflux ratio of 4, equivalent to the 3’-substituted SPA **3** analog. However, avoidance of efflux by the AmTroSPC analogs did not result in an improvement in MIC activity. This is likely due to reduced accumulation within the cell as the result of reduced uptake or potential efflux by the Tap efflux pump homolog (Mab1409c).

### Antimicrobial activity of 3’,6’-disubstituted SPC analogs against other pathogens

The 3’,6’-disubstituted SPC analogs were then tested for antimicrobial activity against a panel of Gram-positive and Gram-negative pathogens (Table 3). TroSPC **2** displayed a considerable increase in activity compared to SPC (>4-fold) against all Gram-positive species with MICs ranging from 4 µg ml^−1^ in *Streptococcus pneumoniae* to 64 µg ml^−1^ against *Staphylococcus aureus* (Table 3). The antimicrobial activity of the analogs was variable across the board. Specifically, the amino TroSPC **11**, TroSPA **4** and the AmTroSPC **13** all had higher MICs in each of the Gram-positive pathogens tested. The bAmSPC **6** and eAmSPC **8** offered modest improvement in activity against several strains, including both *Enterococcus* species, *Staphylococcus epidermidis*, and *Streptococcus pyogenes* when compared to the parent compound. This finding is consistent with previous reports that have highlighted the positive impact of aminomethyl substitutions on potency of SPC analogs against Gram-positive organisms [15]. In contrast, neither TroSPC nor the 3’,6’-disubstituted SPC analogs had any considerable activity against Gram-negative bacteria (MIC > 64 against *Acinetobacter baumannii*, *Klebsiella pneumoniae*, and *Pseudomonas aeruginosa*) outside of *Escherichia coli* species, which was inhibited by bAmSPC **6** and eAmSPC **8** at 32 µg ml^−1^ and 64 µg ml^−1^, respectively (Table 4). All the compounds that were tested displayed reduced MIC values in the *E. coli* ∆*tolC* isogenic mutant, with efflux ratios ranging from ≥2 to 16 (Table 4). This finding supports the notion that efflux, in addition to metabolism by aminoglycoside modifying enzymes, diminishes the susceptibility of the Gram-negatives to synthetic spectinomycins [6, 9].

**Table 3:** Antibacterial susceptibility (MIC, µg ml^−1^) against Gram-positive panel

	Minimum Inhibitory Concentration (µg ml^−1^)					
Compound	*E. faecalis* ATCC 29212	*E. faecium* ATCC 19434	*S. aureus* USA300	*S. epidermidis* ATCC 14990	*S. pneumoniae* R6	*S. pyogenes* ATCC 700294
SPC (**1**)	256	256	512	256–512	16	32
TroSPC (**2**)	64	64	64	32	4	4–8
TroSPA (**4)**	>64	>64	>64	>64	16	8
bAmTroSPC (**6**)	32	32	64	16	4	2
eAmTroSPC **(8)**	32	32	64	8	8	4
Amino TroSPC (**11)**	>64	>64	>64	>64	64	32
AmTroSPC (**13**)	>64	>64	>64	>64	16–32	16

**Table 4:** Antibacterial susceptibility (MIC, µg ml^−1^) against Gram-negative panel

	Minimum Inhibitory Concentration (µg ml^−1^)					
Compound	*A. baumannii* ATCC 19606	*K. pneumoniae* ATCC 700603	*P. aeruginosa* ATCC 15692	*E. coli* K12	*E. coli* K12 ∆*tolC*	*E. coli* Efflux Ratio
SPC (**1**)	>1024	512	1024	128	64	2
TroSPC (**2**)	>64	>64	>64	>64	16	>4
TroSPA (**4)**	>64	>64	>64	>64	32	>2
bAmTroSPC (**6**)	>64	>64	>64	32	4–8	4–8
eAmTroSPC **(8)**	>64	>64	>64	64	4–8	8–16
Amino TroSPC (**11)**	>64	>64	>64	>64	32	>2
AmTroSPC (**13**)	>64	>64	>64	>64	32	>2

## Conclusions

The premise of this study was to determine if combining the 6’-propyl group from TroSPC **2** with the 3’-modifications from our three lead spectinomycin series would result in compounds with more potent antibacterial activity. While this strategy did not improve the MIC activity, it did provide valuable insights in the SAR of the series, improving our overall understanding into the complex relationship between ribosomal inhibition, whole cell activity, and efflux mechanisms of spectinomycins.

In silico experiments illustrated that TroSPC **2** and the disubstituted analogs TroSPA **4** and eAmTroSPC **8** can occupy the helix-34 SPC binding site and maintain critical hydrogen bonding interactions consistent with SPC’s mechanism of action. We were able to generate 3’,6’-disubstituted SPC analogs through modification of previously successful routes for the mono-substituted spectinomycins, although the synthesis is longer and more costly than single substituted analogs. TroSPC **2** and AminoTroSPC **11** displayed similar ribosomal inhibitory properties and whole cell activity against *M. smegmatis* compared to SPC **1**. Modification to the 3’-position present in TroSPA **4** and the AmTroSPCs (**6, 8**, and **13**) results in less potent activity in the ribosome inhibition assay. The decrease in ribosomal inhibition in a cell-free environment, however, does not directly correlate with whole-cell activity. For example, eAmTroSPC **8** demonstrated lower ribosomal IC_50_ values but was among the most potent across all pathogens tested. This discrepancy can be attributed to variations in drug accumulation, which are influenced by differences in efflux mechanisms and uptake rates.

In this study, we found that efflux pumps, such as Rv1258c in *M. tuberculosis*, Mab2780c in *M. abscessus*, and TolC in *E. coli* remain the major contributors to whole cell susceptibility of our compounds. TroSPC was found to be subject to efflux in both *M. tuberculosis* and *M. abscessus*. For the disubstituted compounds, the 3’-modification was found to dominate antimicrobial activity, as it is this modification that is crucial for evading efflux mechanisms. The 6’-propyl was not found to antagonize MIC activity. In *M. tuberculosis*, MIC activity of the disubstituted analogs was similar to their corresponding 3’-substituted analogs in both the wild type and the Rv1258c knockout. The 2-pyridyl TroSPA **4** was shown to evade efflux, while bAmTroSPC **6** and eAmTroSPC **8** were subject to efflux by Rv1258c. Likewise, in wildtype *M. abscessus*, the 3’,6’-disubstituted analogs had similar MIC to the 3’-substituted SPA and AmSPC analogs. Unexpectedly, the AmTroSPC analogs **6** and **8** were able to avoid efflux by TetV. The efflux ratio for these compounds was 1, compared to 16 and 4, for the single 3’-substituted compounds **5** and **7**, respectively. However, avoidance of TetV-mediated efflux did not result in an improved MIC compared to the 3’-modification alone. This is likely due to sacrifices to uptake or additional efflux by the Tap efflux pump homolog Mab1409c, resulting in lower accumulation. This observation leads the way for further modification and SAR analysis with respect to efflux avoidance and understanding resistance mechanisms. The antimicrobial spectrum of activity and potency of these TroSPC analogs closely aligns with that of the 3’-SPC analogs [15, 16, 20]. TroSPA **4** exhibits narrow-spectrum activity against *M. tuberculosis*, while bAmTroSPC **6** displays efficacy against Gram-positive respiratory tract pathogens, and eAmTroSPC **8** provides broad coverage against both Mycobacteria and other pathogens.

A limitation of this study was the use of nutrient-rich conditions and native ribosomes to characterize the newly synthesized TroSPC analogs. It is well documented that under nutrient-limited conditions, Mycobacterial ribosomes undergo significant changes. Zinc starvation induces remodeling of the Mycobacterial ribosome, replacing many ribosomal proteins containing the CXXC zinc-binding motif (C+) with corresponding paralogs lacking this motif (C−) [26]. This is important as these alternative C− proteins are induced during chronic infection and significantly impact translation efficiency and the activity of antimicrobials such as kanamycin, streptomycin, and spectinamides [26–28]. Recent work from the Ojha laboratory demonstrated that spectinamide 1599 had reduced affinity to C− ribosomes, resulting in increased MIC and decreased cell killing in both *M. smegmatis* and *M. tuberculosis* [28]. The reduced activity of SPC was attributed to additional contact points between C− S_14_ ribosomal protein and the 16S rRNA helix 34 binding site, increasing rigidity and reducing rotation of the 30SC− ribosomes [26, 28]. Since the TroSPC analogs described in this study are not predicted to increase binding to 16S rRNA nucleotides (Fig. 2F) and do not improve translational inhibitory activity (Table 2), it is likely that these TroSPC analogs will also have reduced affinity to C− ribosomes compared to C+ ribosomes, similar to their 3’ counterparts. Future discovery efforts should prioritize analogs that overcome this limitation.

This work contributes to the understanding of the SAR of the natural product SPC. Previous and current studies indicate that overcoming innate resistance to spectinomycin involves a balance between ribosomal inhibition, efflux, and uptake, which can be achieved through careful structural modification that addresses these mechanisms collectively. The results of this study will help guide the design of next-generation analogs to overcome drug resistance and improve antimicrobial potency of spectinomycins.
