# Correction: Synthesis and antibacterial action of 3’,6’-disubstituted spectinomycins

**DOI:** 10.1038/s41429-024-00777-5

**Published:** 2024-10-11

**Authors:** Suresh Dharuman, Gregory A. Phelps, Christine M. Dunn, Laura A. Wilt, Patricia A. Murphy, Robin B. Lee, Hannah E. Snoke, Petra Selchow, Klara Haldimann, Erik C. Böttger, Sven N. Hobbie, Peter Sander, Richard E. Lee

**Affiliations:** 1 Department of Chemical Biology and Therapeutics, St. Jude Children’s Research Hospital, 262 Danny Thomas Place, MS#1000, Memphis, TN 38105 USA; 2 Graduate School of Biomedical Sciences, St. Jude Children’s Research Hospital, Memphis, TN 38103 USA; 3 Institute of Medical Microbiology, University of Zurich, Gloriastrasse 28/30, CH-8006 Zurich, Switzerland; 4 Division of Clinical Bacteriology and Mycology, University Hospital Basel, Petersgraben 4, CH-4031 Basel, Switzerland; 5 National Reference Center for Mycobacteria, Gloriastrasse 28/30, CH-8006 Zurich, Switzerland

**Keywords:** Antibiotics, Drug discovery and development

Correction to: *The Journal of Antibiotics*

10.1038/s41429-024-00750-2 published online 18 June 2024

In this article, affiliation to Division of Clinical Bacteriology and Mycology, University Hospital Basel was mistakenly attributed to Erik C. Böttger instead of Sven N. Hobbie.

The original article has been corrected.

